# Biology and Epidemiology of *Venturia* Species Affecting Fruit Crops: A Review

**DOI:** 10.3389/fpls.2017.01496

**Published:** 2017-09-19

**Authors:** Elisa González-Domínguez, Josep Armengol, Vittorio Rossi

**Affiliations:** ^1^Department of Sustainable Crop Protection, Università Cattolica del Sacro Cuore Piacenza, Italy; ^2^Instituto Agroforestal Mediterráneo, Universitat Politècnica de València Valencia, Spain

**Keywords:** *Fusicladium* spp., *Spilocaea* spp., fruit scab, integrated pest management, multiple correspondence analysis

## Abstract

The fungal genus *Venturia* Sacc. (anamorph *Fusicladium* Bonord.) includes plant pathogens that cause substantial economic damage to fruit crops worldwide. Although *Venturia inaequalis* is considered a model species in plant pathology, other *Venturia* spp. also cause scab on other fruit trees. Relative to the substantial research that has been conducted on *V. inaequalis* and apple scab, little research has been conducted on *Venturia* spp. affecting other fruit trees. In this review, the main characteristics of plant-pathogenic species of *Venturia* are discussed with special attention to *V. inaequalis* affecting apple, *V. pyrina* affecting European pear, *V. nashicola* affecting Asian pear, *V. carpophila* affecting peach and almond, *Fusicladium oleagineum* affecting olive, *F. effusum* affecting pecan, and *F. eriobotryae* affecting loquat. This review has two main objectives: (i) to identify the main gaps in our knowledge regarding the biology and epidemiology of *Venturia* spp. affecting fruit trees; and (ii) to identify similarities and differences among these *Venturia* spp. in order to improve disease management. A thorough review has been conducted of studies regarding the phylogenetic relationships, host ranges, biologies, and epidemiologies of *Venturia* spp. A multiple correspondence analysis (CA) has also been performed on the main epidemiological components of these *Venturia* spp. CA separated the *Venturia* spp. into two main groups, according to their epidemiological behavior: the first group included *V. inaequalis, V. pyrina, V. nashicola*, and *V. carpophila*, the second *F. oleagineum* and *F. eriobotryae*, with *F. effusum* having an intermediate position. This review shows that *Venturia* spp. affecting fruit trees are highly host-specific, and that important gaps in understanding the life cycle exist for some species, including *V. pyrina*; gaps include pseudothecia formation, ascospore and conidia germination, and mycelial growth. Considering the epidemiological information reviewed, this paper shows that the use of Mills tables to predict infection periods should be avoided for *Venturia* spp. other than *V. inaequalis*.

## Introduction

The fungal genus *Venturia* Sacc. (anamorph *Fusicladium* Bonord.) includes plant pathogens that cause substantial economic damage to fruit crops worldwide (Sivanesan, 1977; Schubert et al., 2003). Although, *Venturia inaequalis* (Cooke) G. Winter is considered a model species in plant pathology (Machardy, 1996) and it is the causal agent of apple scab, the most important apple disease worldwide, other *Venturia* spp. also cause scab on other fruit trees. In this review, the main characteristics of plant-pathogenic species of *Venturia* are discussed with special attention to *V. inaequalis* affecting apple (*Malus* spp.); *V. pyrina* and *V. nashicola* affecting European pear (*Pyrus communis*) and Asian pear (*P. pyrifolia* var. *culta* and *P. ussuriensis*), respectively; *V. carpophila* affecting peach (*Prunus domestica*) and almond (*Prunus dulcis*); *Fusicladium oleagineum* affecting olive (*Olea europea*); *F. effusum* affecting pecan (*Carya illinoinensis*); and *F. eriobotryae* affecting loquat (*Eriobotrya japonica*). Aspects of *V. asperata* affecting apple and *V. cerasi* affecting cherry (*Prunus cerasus*) are also discussed (Table 1).

**Table 1:** Species of *Venturia* included in this review.

**Pathogen^a^**	**Host^b^**	**Sexual phase/deciduous tree**	**Authority**	**Principal synonyms**
***Fusicladium effusum***	*Carya illinoinensis* (Wangenh.) K. Koch	−/−	G. Winter (1885)	*Cladosporium effusum*; *C. caryigenum*
***Fusicladium eriobotryae***	*Eriobotrya japonica* (Thunb.) Lindl.	−/−	(Cavara) Sacc. (1892)	*Spylocaea. pyracanthae*; *S. eriobotryae*; *F. pyracanthae*
***Fusicladium oleagineum***	*Olea europaea* L.	−/−	Ritschel & U. Braun (2003)	*S. oleaginea*; *Cycloconium oleagineum*
***Fusicladium pyracanthae***	*Pyracantha* spp.	−/−	(Thüm.) O. Rostr. (1912)	*S. pyracanthae*; *F. eriobotryae; F. pyrorum var. pyracanthae*;
***Venturia asperata**/Fusicladium asperatum*	*Malus* spp.	+/+	Samuels & Sivan (1975)	
***Venturia carpophila*****/***Fusicladium carpophilum*	*Prunus domestica* L./*Prunus dulcis* (Mill.) D.A.Webb	+/+	E.E. Fisher (1961)	*C. carpophilum*; *Fusicladosporium carpophilum*
***Venturia cerasi/**Fusicladium cerasi*	*Prunus cerasus* L.	+/+	Aderh. (1900)	*V. chlorospora; Acrosporium cerasi; C. cerasi, Megacladosporium cerasi*
***Venturia inaequalis*****/***Fusicladium pomi*	*Malus* spp.	+/+	(Cooke) G. Winter (1875)	*F. denditricum*; *S. pomi; Cladosporium denditricum*
***Venturia nashicola*****/***Fusicladium nashicola*	*Pyrus pyrifolia* Nakai var. *culta* Nakai/*P. ussuriensis* Maxim.	+/+	S. Tanaka & S. Yamam. (1964)	
***Venturia pyrina*****/***Fusicladium pyrorum*	*Pyrus communis* L.	+/+	Aderh. (1896)	*V. pirina; Helminthosporium pyrorum*; *V. pyrina* f. sp. *piri*

a *Names currently accepted (in bold); for pleomorphic species, names of the anamorphs are indicated after the slash*.

b *Based on Sivanesan (1977) and Schubert et al. (2003)*.

**Figure 1:**
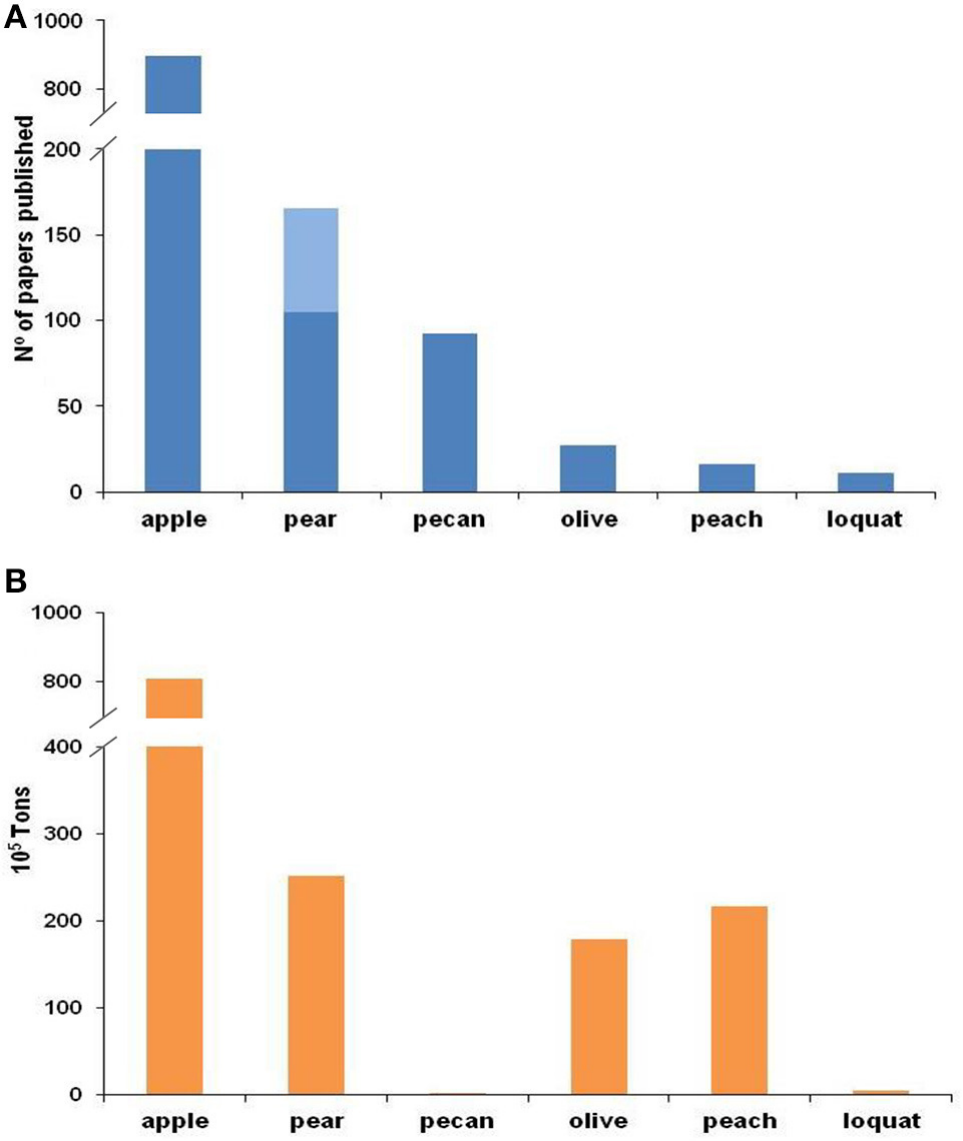
Number of papers published on scab (A) and worldwide production (B) for each fruit crop. For **(A)**, the Web of Science was searched on 30 August 2016 for different combinations of words in the title (for apple: “*Venturia inaequalis”* or “*Fusicladium pomi*” or “*Spilocaea pomi*” or “apple scab”; for pear: “*Venturia pyrina*” or “*Venturia pirina*” or “*Fusicladium pyrorum*” or “pear scab” and in light blue “*Venturia nashicola*” or “*Fusicladium nashicola*”; for pecan: “*Fusicladium effusum*” or “*Cladosporium effusum*” or “*Cladosporium caryigenum*” or ”pecan scab”; for olive “*Spilocaea oleagina*” or “*Fusicladium oleagineum*” or “olive scab” or “olive leaf spot”; for peach: “*Venturia carpophila*” or “*Fusicladium carpophilum*” or “*Cladosporium carpophilum*” or “peach scab”; and for loquat: “*Fusicladium eriobotryae*” or “*Spilocaea eriobotryae*” or “loquat scab”). Worldwide production data for **(B)** were extracted from FAOSTAT, except in the case of loquat, where data from González-Domínguez (2014) were used.

Since the late nineteenth century, apple scab has been extensively investigated, and substantial information—covering all key aspects of the biology and genetics of the fungus and the epidemiology and control of the disease—has been published and reviewed by Machardy (1996) and Bowen et al. (2011). In contrast to the efforts devoted to investigating *V. inaequalis*, little work has been conducted on *Venturia* spp. affecting other fruit trees, as shown by the number of papers published for each species (Figure 1A). This difference in research effort and number of publications, however, does not directly reflect the importance of the host crops worldwide (Figure 1B). The difference might be explained by (i) minor investments in these non-apple crops, (ii) less specialized management directed at the non-apple crops, and (iii) the common use of the information developed for *V. inaequalis* for managing the other fruit scabs. Concerning the last point, researchers generally assume that infection of any scab fungus may occur under environmental conditions similar to those required by *V. inaequalis*. The Mills and Laplante's (1954) table, which is the most popular system for scheduling fungicides against apple scab, has been broadly recommended for management of pear scab (Sobreiro and Mexia, 2000; Mitcham and Elkins, 2007; Travis et al., 2012; Elkins et al., 2016), cherry scab (Schweizer, 1958), peach scab (Keitt, 1917; Pineau et al., 1991), and loquat scab (Ramos, 2008; GVA, 2014). However, there is no clear evidence that the environmental conditions conducive for infection are similar for all of these *Venturia* species. In fact, recent studies have revealed important differences concerning the environmental requirements for infection by *F. eriobotryae* and *F. oleagineum* vs. *V. inaequalis* (Viruega et al., 2011; González-Domínguez et al., 2013). In addition, substantial differences exist in the ecophysiologies and the life cycles of their hosts.

This review has two main objectives: (i) to identify the main gaps in our knowledge regarding the biology and epidemiology of *Venturia* spp. affecting fruit trees; and (ii) to identify similarities and differences between these *Venturia* spp. in order to improve disease management. To accomplish these objectives, the authors have thoroughly reviewed the studies regarding the phylogenetic relationships, host ranges, biologies, and epidemiologies of *Venturia* spp. Finally, the review discusses the implications of the similarities and differences in the fungi and the diseases for the management of the different scab diseases.

## Taxonomy of the genus *Venturia*

The genus *Venturia* belongs to the phylum Ascomycota, class Dothideomycetes (Schoch et al., 2009). Traditionally, this genus has been included in the family Venturiaceae, order Pleosporales, according to its “Pleospora-type centrum and bitunicate asci” (Sivanesan, 1977). However, recent molecular phylogenetic analyses of Dothideomycetes, using both nuclear and mitochondrial gene regions, have indicated that the family Venturiaceae forms a well-supported monophyletic group separate from the Pleosporales (Kodsueb et al., 2006; Kruys et al., 2006; Zhang et al., 2011). Thus, Zhang et al. (2011) recently reordered Venturiaceae into Venturiales ord. nov. (together with Sympoventuriaceae fam. nov.).

The genus *Venturia* Sacc. (1882) was first noted in 1844 by Notaris, who described, *V. dianthi* and *V. rosae*. Cesati & Notaris added new species in 1863 (Sivanesan, 1977). Saccardo reevaluated the genus in 1882, excluding both *V. dianthi* and *V. rosae* (Sivanesan, 1977). Sivanesan (1977) listed 52 species of *Venturia*, which comprised parasitic species with pseudothecia immersed in the host tissue, bitunicate asci, and olive-brown, septate ascospores (Sivanesan, 1977). Currently, 290 species are recognized in the Mycobank dabatase (http://www.mycobank.org/Biolomics.aspx?Table=Mycobankx, March/2017).

The anamorphs of *Venturia* spp. have been traditionally classified in three genera: *Fusicladium* Bonord., *Pollaccia* Baldacci & Cif., and *Spilocaea* Fr. Assignment to these genera depends on the morphology of the conidiogenous cells. These cells are sympodial in *Fusicladium* and percurrent in *Pollaccia* and *Spilocaea* (Hughes, 1953; Sivanesan, 1977). Recents works that used both morphological and molecular characters concluded that the anamorphic species of *Venturia* should not be separated into these three genera because (i) most species have both sympodial and percurrent conidiogenous cells, and (ii) molecular phylogenetic analysis clearly shows that *Venturia* and its anamorphs are monophyletic group (Schubert et al., 2003; Beck et al., 2005). Because most anamorphs of *Venturia* have been classified as *Fusicladium*, this name was proposed to designate the asexual stage of *Venturia* spp. (Braun et al., 2002), and used in the monograph written by Schubert et al. (2003). Very recently, the International Commission on the Taxonomy of Fungi has proposed the use of *Venturia* instead of *Fusicladium* for the species with only anamorph stage, following the guidelines of the “Amsterdam Declaration on Fungal Nomenclature” (May, 2017). In this paper we keep the dual nomenclature because it facilitates to distinguish the species with or without sexual phase.

In 2003, Partridge and Morgan-Jones (2003) proposed the new genus *Fusicladosporium*, including the anamorphs of pecan (*F. effusum*), peach (*F. carpophillum*), and maple scab (*F. humile*; teleomorph *V. acerina*). The authors considered that significant morphological differences (conidia formed in chains and the prominence of conidial scars on conidiophores) distinguished these anamorphs from those in the genera *Cladosporium* and *Fusicladium*. However, this new taxon seems unjustified because two older generic names for *Venturia* anamorphs with catenate conidia are available (*Hormocladium* Höhn. and *Ramalia* Bat) and because phylogenetic analysis of the ITS region demonstrates that the erection of *Fusicladosporium* results in a polyphyletic genus (Schubert et al., 2003; Beck et al., 2005). Thus, *Fusicladosporium* is currently considered a synonym of *Fusicladium* (Schubert et al., 2003; Crous et al., 2007; Scherm et al., 2008; Seyran et al., 2009; Lalancette et al., 2012).

## *Venturia* species as fruit tree pathogens

### Geographical distribution

**Figure 2:**
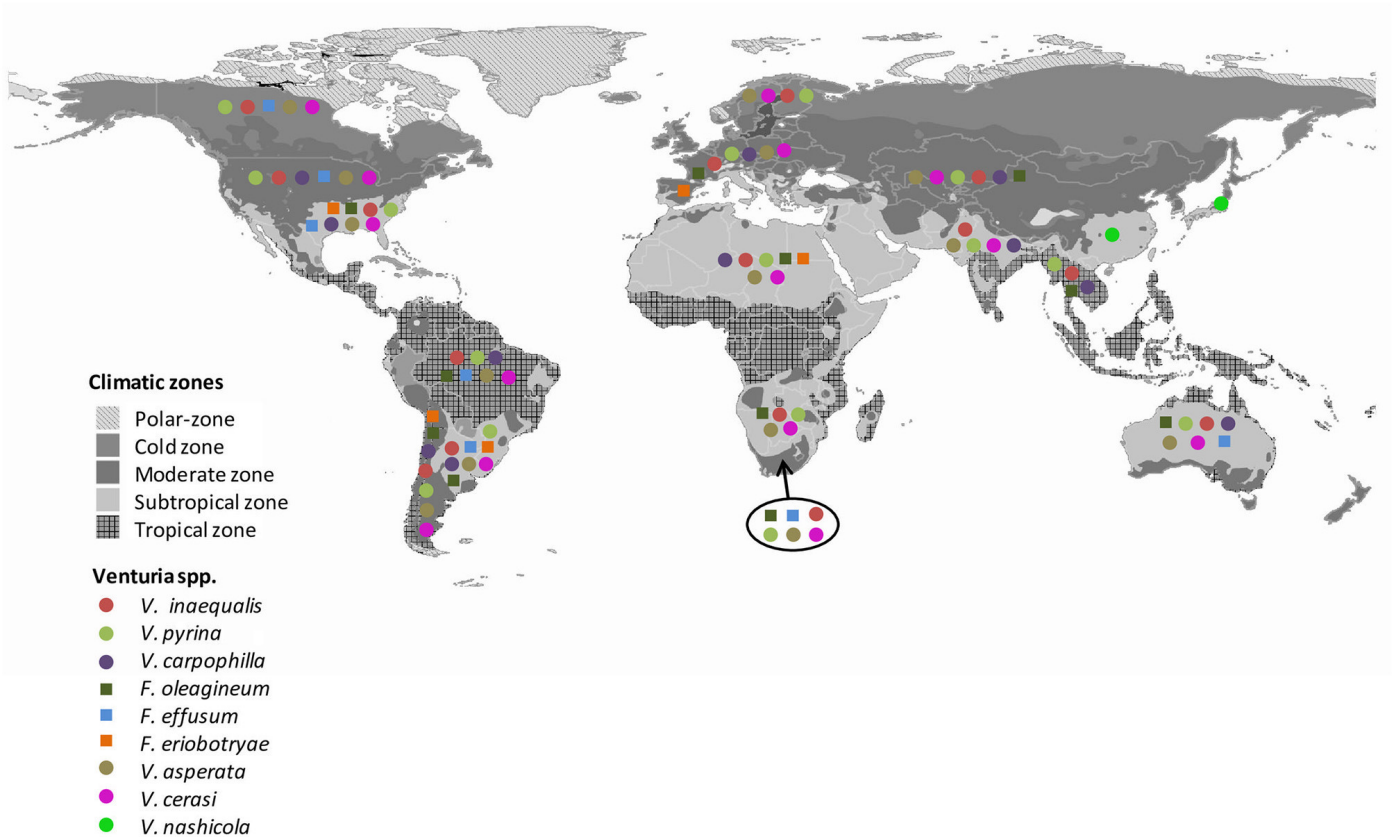
Worldwide distribution of *Venturia* spp. Gray colors indicate the climate regions proposed by Kottek et al. (2006). Colors and symbols indicate the presence of the different *Venturia* spp. in each region. Data are from Plantwise and EPPO database.

Differences exist in the geographical distribution of the *Venturia* spp. that are pathogens of fruit trees (Figure 2).

There are numerous reports about the worldwide distribution of apple scab (Machardy, 1996; Schnabel et al., 1999; Rossi et al., 2007; Gladieux et al., 2008, 2010b; Xu et al., 2009; Bowen et al., 2011; Li et al., 2011; Padder et al., 2013). Apple scab occurs in every country where apple (*Malus x domestica*) is cultivated (Machardy, 1996; Gladieux et al., 2008), with the exception of West Australia, where the disease was eradicated (McKirdy et al., 2001). *Venturia inaequalis* probably emerged in Central Asia, the center of apple origin (Tenzer and Gessler, 1999; Gladieux et al., 2008, 2010b; Xu et al., 2008, 2012), and followed its host's expansion into Europe and, more recently, into regions with the expansion of apple cultivation (Gladieux et al., 2008). *Venturia inaequalis* infecting apple in Europe and Central Asia consists of three distinct populations: (i) a large European population infecting the domesticated apple and the wild *Malus sylvestris*; (ii) a large Central Asian population infecting the domesticated apple and populations of *Malus sieversii*; and (iii) a more geographically restricted population associated with *M. sieversii* in areas where *M. domestica* is absent (Gladieux et al., 2010b). Xu et al. (2008, 2012) found a higher variability in a population of *V. inaequalis* from the same orchard in the UK than in populations from different cultivars or regions in China. Overall, *V. inaequalis* appears to be a model invasive plant pathogen with a broad geographic distribution and well-established populations (Gladieux et al., 2010b).

Like *V. inaequalis, V. pyrina* has a worldwide distribution that is closely associated with the distribution of its host, the European pear (*P. communis*; Ogawa and English, 1991; Figure 2). *V. nashicola*, in contrast, is restricted to China, Japan, South Korea, and Taiwan, where Japanese and Chinese pears are widely cultivated (www.plantwise.org; EPPO, 2016). *V. nashicola* is currently considered a quarantine organism in the EU, USA, Israel, and Turkey (EPPO, 2016).

*Venturia carpophila* affecting *Prunus* spp., *F. oleagineum* affecting olive, and *V. cerasi* affecting cherries have relatively restricted distributions, which again reflects the distributions of their hosts (www.plantwise.org) (Figure 2). *Venturia cerasi* has been reported in Canada, Brazil, New Zealand, Iran, and northern Europe, where cherry is widely cultivated (www.plantwise.org; www.fao.org). *Fusicladium oleagineum* is widespread in the Mediterranean basin as well as in other temperate and subtropical areas of the world (Graniti, 1993). However, its presence has not been noted in other areas where olives are grown, such as North America, Northern Europe, and South Asia (i.e., India, Nepal, Thailand, and Vietnam; Figure 2). The distribution of *V. carpophila* is similar to that of *F. oleagineum*, although the former species has been reported in South Asia but not in South Africa (www.plantwise.org).

*Fusicladium effusum* and *F. eriobotryae* apparently have relatively restricted distributions. *F. effusum* has been detected in South Africa and Australia (Figure 2) and is widely distributed in the Americas, where pecan is an important crop. *F. eriobotryae* has mainly been reported in the Mediterranean basin, but it was also reported in the USA and Chile (Raabe and Gardner, 1972; Acuña, 2010). As far as known, *F. eriobotryae* is not present in China, the center of origin of loquat. Moreover, no references were found confirming the presence of *F. eriobtryae* in Japan or Taiwan, where loquat is widely cultivated.

### Phylogenetic relationships

Several molecular studies have evaluated the phylogenetic relationships among the *Venturia* species affecting fruit trees (Schnabel et al., 1999; Stehmann et al., 2001; González-Lamothe et al., 2002; Le Cam et al., 2002; Beck et al., 2005; Sánchez-Torres et al., 2009; Gladieux et al., 2010a; Zhao G.-J. et al., 2011; Zhao P. et al., 2011). In these studies, *Venturia* species and their anamorphs formed a monophyletic clade composed of several small sub-clades. The sub-clades mainly contain strains of the same species, although in some cases strains from different species form a unique sub-clade, as occurs with *V. cerasi* and *V. asperata* (Schnabel et al., 1999; Stehmann et al., 2001; González-Lamothe et al., 2002; Beck et al., 2005), *V. pyrina* and *V. nashicola* (Schnabel et al., 1999; Stehmann et al., 2001; González-Lamothe et al., 2002; Beck et al., 2005), and *V. inaequalis, F. eriobotryae*, and *F. pyracanthae* (Le Cam et al., 2002). All of these analyses concerned the ITS region, which is the most widely used region for species identification in mycology. However, results from this region cannot be considered conclusive for distinguishing closely related fungal species (Kiss, 2012; Schoch et al., 2012). When other nuclear loci have been used, such as the elongation factor or the G3PD genes, differences between closely related *Venturia* spp. have been observed (Sánchez-Torres et al., 2009; Gladieux et al., 2010b; Zhao P. et al., 2011).

*V. inaequalis, F. eriobotryae*, and *F. pyracanthae* exhibit a high sequence similarity in their ITS and other DNA regions (Gladieux et al., 2010a). In general, the topology of the phylogram for *Venturia* species aligns closely with that of the host genera, demonstrating a close co-evolutionary relationship between the pathogenic *Venturia* spp. and their respective fruit tree hosts (Ishii and Yanase, 2000; Beck et al., 2005; Bowen et al., 2011). Because of these high similarity and based on the criterion of concordance between multiple gene genealogies (Taylor et al., 2000), Le Cam et al. (2002) and Gladieux et al. (2010a) considered *F. eriobotryae* and *F. pyracanthae* as *formae speciales* of *V. inaequalis*. Sánchez-Torres et al. (2009) performed further molecular analyses (a phylogenetic analysis of the G3PD gene, a microsatellite-primed PCR analysis, and RAPD fingerprinting) and pathogenicity tests for *F. eriobotryae* and *V. inaequalis*; these authors concluded that the loquat scab fungus is a distinct species from *V. inaequalis*.

Similarly, researchers were able to distinguish between *V. pyrina* and *V. nashicola* based on a further phylogenetic analysis of the elongation factor gene (Zhao P. et al., 2011), whereas an earlier study was unable to do so based on the ITS region (Beck et al., 2005). Until the 1960s, *V. pyrina* was considered the causal agent of scab on Japanese and Chinese pear (*Pyrus pyrifolia* var. *culta* and *P. ussuriensis*, respectively). However, further studies indicated that the causal agent of scab on Asian pears differed from *V. pyrina* (Tanaka and Yamamoto, 1964; Ishii and Yanase, 2000; Abe et al., 2008; Zhao P. et al., 2011).

The results described in the previous paragraphs show that *Venturia* spp. have a monophyletic evolutionary origin and a close co-evolutionary development with their hosts. In addition, species delimitations become clearer when the phylogenetic analyses are performed in DNA regions in addition to the ITS region.

### Host range

*Venturia* spp. are confined to six host families: Acaraceae, Betulaceae, Cornaceae, Oleaceae, Rosaceae, and Salicaceae (Sivanesan, 1977). *Venturia* spp. also seem to be highly host specific in that each species is usually confined to one host genus or at least to allied host genera in one host family (Schubert et al., 2003).

Some controversy exists regarding the host range of the *Venturia* spp. on fruit trees. In the monograph of *Venturia* published in 1977, Sivanesan listed 14 species of Rosaceae as hosts of *V. inaequalis*, and these hosts belonged to the genera *Cotoneaster, Malus, Pyracantha, Pyrus*, and *Sorbus*. Schubert et al. (2003) increased the hosts of *V. inaequalis* to including 12 genera by adding *Amelanchier, Aronia, Docynia, Eriobotrya, Heteromeles, Kageneckia*, and *Prunus*. The latter authors, however, did not cite specific studies regarding the ability of *V. inaequalis* to infect these hosts, and some of these host-pathogen interactions have been refuted (Menon, 1956; Raabe and Gardner, 1972; Ishii and Yanase, 2000; Stehmann et al., 2001; Le Cam et al., 2002; Chevalier et al., 2004; Sánchez-Torres et al., 2007a, 2009; Abe et al., 2008).

Both Schubert et al. (2003) and Sivanesan (1977) considered *P. communis* to be a host of *V. inaequalis*; Schubert et al. (2003) also considered *Malus domestica* to be a host of *V. pyrina*. Other studies, however, have failed to obtain infection of pear by *V. inaequalis* or infection of apple by *V. pyrina* (Menon, 1956; Stehmann et al., 2001; Chevalier et al., 2004) (Table 2). Menon (1956) inoculated apple and pear plants with three kinds of *V. inaequalis* and *V. pyrina* inocula (mycelium plugs, conidial suspensions, and ascospore suspensions) and observed clear scab symptoms on apple only with *V. inaequalis* and on pear only with *V. pyrina* and in both cases only with conidia or ascospores (Table 2).

**Table 2:** Results of cross inoculations of *Venturia* spp. on different Rosaceous hosts.

***Venturia* species**	**Host species**						
	**Apple (*Malus domestica*)**	**European pear (*Pyrus communis*)**	**Japanese pear (*P. pyrifolia* var. *culta*)**	**Chinese pear (*P. ussuriensis*)**	**Blackthorn (*Prunus spinosa*)**	**Pyracantha (*Pyracantha* spp.)**	**Loquat (*Eriobotrya japonica*)**
*V. inaequalis*	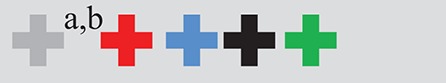	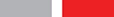			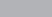	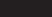	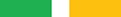
*V. pyrina*	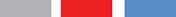	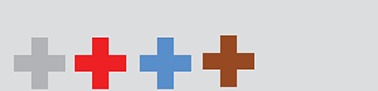	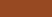	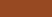	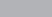		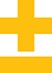
*V. nashicola*		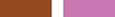	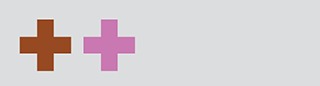	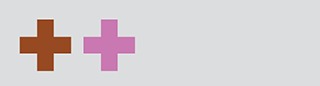			
*V. cerasi*	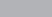	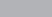	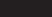	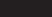	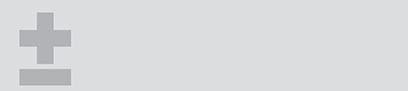		
*F. pyracanthae*	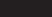					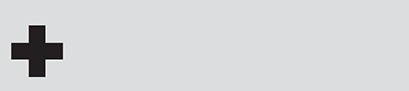	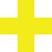
*F. eriobotryae*							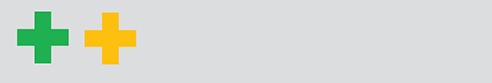
*V. carpophila*							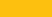

*^a^+ indicates a successful infection, −indicates the absence of symptoms, ± indicates that infection was uncertain. Gray area indicates the standard host for each species*.

*^b^Different colors refer to the references in which the pathogenicity experiments were published (gray = Menon, 1956; red = Chevalier et al., 2004; blue = Stehmann et al., 2001; black = Le Cam et al., 2002; green = Sánchez-Torres et al., 2007a; orange = Sánchez-Torres et al., 2009; yellow = Raabe and Gardner, 1972; brown = Ishii and Yanase, 2000; pink = Abe et al., 2008). In Menon (1956), V. cerasi was isolated from Prunus padus*.

Stehmann et al. (2001) found pseudothecia of both *V. inaequalis* and *V. pyrina* in dead apple leaves but cross inoculations with the conidial suspensions of these isolates on detached apple and pear leaves resulted in infection only when apple was inoculated with *V. inaequalis* and when pear was inoculated with *V. pyrina* (Table 2). Although the conidia of *V. inaequalis* and *V. pyrina* germinated on both hosts, and although the germ tubes produced appressoria and runner hyphae on both hosts, a dense subcuticular network of stroma was produced only in the compatible host (Stehmann et al., 2001; Chevalier et al., 2004).

Pyracantha (*Pyracantha* spp.) and loquat (*E. japonica*) have also been considered hosts of *V. inaequalis* (Sivanesan, 1977; Jones and Aldwinckle, 1990; Machardy, 1996; Schubert et al., 2003; Jha et al., 2009; Bowen et al., 2011). However, Le Cam et al. (2002) were unable to obtain infection of pyracantha by *V. inaequalis* or infection of apple by *F. pyracanthae* (Table 2). Similarly, inoculations of loquat with *V. inaequalis, V. pyrina*, or *V. carpophila* did not cause infection (Sánchez-Torres et al., 2007b, 2009; Table 2). Loquat plants inoculated with *V. pyrina* showed symptoms on leaves, but the symptoms differed from those caused by the loquat scab fungus *F. eriobotryae* (Sánchez-Torres et al., 2007b). Raabe and Gardner (1972) successfully infected loquat plants with *F. pyracanthae*. Based on this information, Gladieux et al. (2010a) considered *F. pyracanthae* and *F. eriobotryae* as unique species, but the results of Raabe and Gardner (1972) have never been confirmed.

The designation of *V. nashicola* and *V. pyrina* as distinct species was confirmed by the unsuccessful inoculation of European pear (*P. communis*) by *V. nashicola* and of Japanese and Chinese pear (*P. pyrifolia* and *P. ussuriensis*, respectively) by *V. pyrina* (Ishii and Yanase, 2000; Park et al., 2000; Abe et al., 2008). Like *V. inaequalis* and *V. pyrina* on non-hosts, *V. nashicola* germinated and formed appressoria on European pears but the hyphae collapsed after the host was penetrated (Abe et al., 2008).

Overall, results from cross inoculations of *Venturia* spp. on different Rosaceous hosts (Table 2) show that these pathogens are host specific, and the information contained in the monographs of Schubert et al. (2003) and Sivanesan (1977) cannot be reproduced. Host specificity requires further study for *F. pyracanthae* and *F. eriobotryae*.

### Life cycle

A main characteristic distinguishing the life cycles of some *Venturia* spp. from others is the presence/absence of the sexual stage (Table 1). *Venturia inaequalis, V. pyrina, V. nashicola*, and *V. cerasi* form pseudothecia in detached leaves on the orchard ground (Schweizer, 1958; Latorre et al., 1985; Umemoto, 1990b; Spotts and Cervantes, 1994; Machardy, 1996; Rossi et al., 2001; Eguchi and Yamagishi, 2007; Lian et al., 2007). Pseudothecia of *V. carpophila* were also observed in affected detached leaves of apricot (Fisher, 1961), but their epidemiological role is unknown, and the conidia of *V. carpophila* overwintering in affected twigs are commonly considered the primary inoculum (Lan and Scherm, 2003; Lalancette et al., 2012). Ascocarps have never been found in nature for *F. effusum, F. oleagineum, F. eriobotryae*, or *F. pyracanthae*; conidia are considered the only form of primary inoculum for these species (Raabe and Gardner, 1972; Gottwald and Bertrand, 1982; Graniti, 1993; González-Domínguez et al., 2014b).

Interestingly, pseudothecia are found in those *Venturia* spp. affecting deciduous fruit trees such as apple, cherry, nectarine, or European and Asian pears, but not in those species affecting evergreen trees, including pyracantha, loquat, and olive (Table 1).

**Figure 3:**
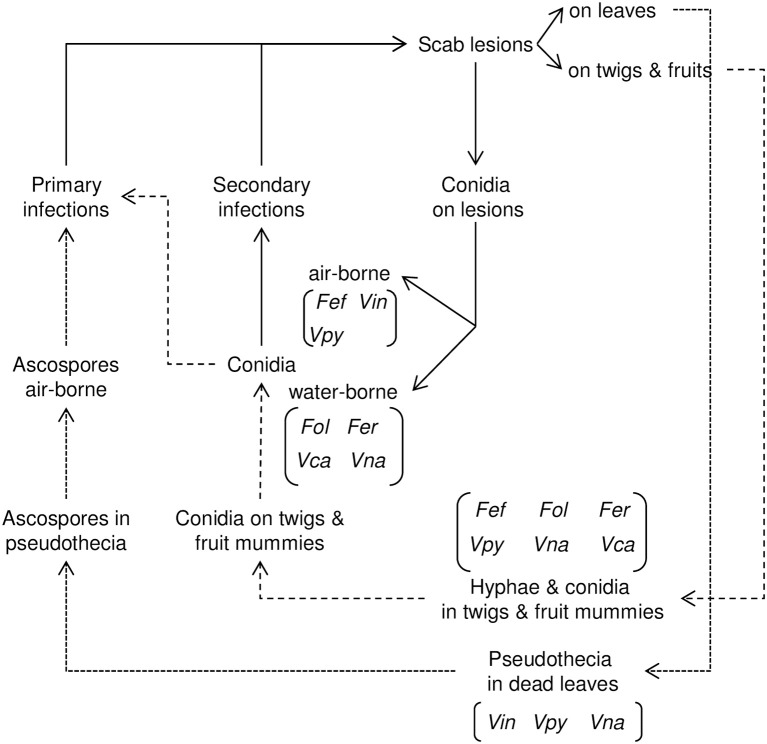
Relational diagram of the life cycle of *Venturia* spp. Dotted lines (…) indicate the sexual phase of the life cycle; dashed lines (-----) indicate the asexual phase of the life cycle. The species of *Venturia* in which the different stages occur are indicated in brackets. Fef, *Fusicladium effusum*; Fer, *Fusicladium eriobotryae*; Fol, *Fusicladium oleagineum*; Vca, *Venturia carpophila*; Vin, *Venturia inaequalis*; Vna, *Venturia nashicola*; Vpy, *Venturia pyrina*.

The pseudothecia-forming species survive winter mainly as pseudothecia in detached leaves on the surface of the orchard floor. In spring, these pseudothecia repeatedly discharge ascospores, which constitute the primary inoculum for infection. Species that lack pseudothecia overwinter (or oversummer) as mycelium and conidia in lesions on twigs and leaves and in the mummified fruits remaining in the tree after harvest (Figure 3).

The asexual cycle is similar for all *Venturia* species. Conidia produced on lesions cause secondary infections during the entire tree-growing season as long as the environmental conditions permit conidial production, dispersal, germination, infection, and lesion growth (Figure 3).

### Main epidemiological components

#### Primary inoculum sources

*Venturia inaequalis* survives in winter mainly as pseudothecia on dead, scabbed leaves on the ground (Table 3; Machardy, 1996). Under some environmental conditions, the fungus can survive in winter as conidia, and when this occurs, these conidia contribute to the primary inoculum. Studies by Holb et al. (2004a) in The Netherlands, Hill (1975) in Germany, and Becker et al. (1992) in New York found that, although large numbers of conidia are present on the surface of shoots or outer bud tissues, conidia are able to overwinter (i.e., are viable in early spring) only on the inner of bud tissues. Becker et al. (1992) and Holb et al. (2004a) reported that this conidia that overwinter in buds are able to infect young green tissues. Recently, Passey et al. (2017) suggested that conidia may contribute 20–50% of the primary inoculum in early spring.

**Table 3:** Publications reporting specific experiments concerning the epidemiological components of *Venturia* spp.

**Epidemiological components**	***Venturia* species**						
	***V. inaequalis***	***V. pyrina***	***V. nashicola***	***V. carpophila***	***F. effusum***	***F. oleagineum***	***F. eriobotryae***
Primary inoculum sources	Hill, 1975; Becker et al., 1992; Machardy, 1996; Holb et al., 2004b; Passey et al., 2017	Marsh, 1933; Kienholz and Childs, 1937; Williamson and Burchill, 1974; Bearden et al., 1976; Latorre et al., 1985; Spotts et al., 2000; Rossi et al., 2009	Li, 1959; Yin and Yu, 1988; Umemoto, 1990b; Lian et al., 2006	Fisher, 1961; Scherm et al., 2008; Lalancette et al., 2012	Demaree, 1924	Graniti, 1993; Viruega et al., 2013	–
Pseudothecia formation and ascospore maturation	Wilson, 1928; Holz, 1937; Hirst and Stedman, 1962; O'Leary and Sutton, 1986; Machardy, 1996	–	Lian et al., 2006	–	–	–	–
Ascospore discharge and dispersal	Aylor and Anagnostakis, 1991; Machardy, 1996; Stensvand et al., 1998; Holb et al., 2004b	Latorre et al., 1985; Spotts and Cervantes, 1994; Villalta et al., 2001; Rossi et al., 2009	Umemoto, 1990a; Eguchi and Yamagishi, 2007; Lian et al., 2007	–	–	–	–
Ascospore germination	Louw, 1948; Boric, 1985	–	Lian et al., 2007	–	–	–	–
Ascospore infection	Keitt and Jones, 1926; Mills, 1944; Machardy and Gadoury, 1989; Machardy, 1996; Stensvand et al., 1997	Villalta et al., 2000	–	–	–	–	–
Production of conidia	Studt and Weltzien, 1975; Machardy, 1996	Ben-Yephet, 1977	–	Lawrence and Zehr, 1982; Lalancette et al., 2012	–	Obanor, 2006	Marras, 1963
Dispersal of conidia	Frey and Keitt, 1925; Wiesmann, 1932; Hirst and Stedman, 1962; Machardy, 1996; Holb et al., 2004a	Kienholz and Childs, 1937	Umemoto, 1990a	Lawrence and Zehr, 1982; Gottwald, 1983; Lan and Scherm, 2003	Gottwald, 1982; Gottwald and Bertrand, 1982; Latham, 1982	Lops et al., 1993; Viruega et al., 2013	González-Domínguez et al., 2014b
Germination of conidia	Boric, 1985; Machardy, 1996	–	Li et al., 2003	Lawrence and Zehr, 1982	Converse, 1956	Obanor et al., 2007	González-Domínguez et al., 2013
Infection by conidia	Machardy and Gadoury, 1989; Machardy, 1996	Spotts and Cervantes, 1994; Villalta et al., 2000	Li et al., 2003, 2005	Scherm and Brannen, 2004	Gottwald, 1985	Obanor et al., 2010; Viruega et al., 2011	Sánchez-Torres et al., 2009; González-Domínguez et al., 2013
Mycelial growth	Machardy, 1996	–	–	Lawrence and Zehr, 1982	–	–	González-Domínguez et al., 2013
Latency period	Mills, 1946; Tomerlin and Jones, 1982	–	–	–	–	Viruega et al., 2011; Roubal et al., 2013	–

*Venturia pyrina* overwinters as both pseudothecia in affected leaves on the orchard floor and as conidia in twig lesions (Marsh, 1933; Kienholz and Childs, 1937; Williamson and Burchill, 1974; Bearden et al., 1976; Latorre et al., 1985; Spotts et al., 2000; Rossi et al., 2009). Based on conidia and ascospore trappings in England and Oregon, respectively, Marsh (1933) and Kienholz and Childs (1937) considered that primary infections by *V. pyrina* resulted largely from conidia derived from the previous seasons. Other reports have indicated that ascospores from pseudothecia are the main inoculum source (Bearden et al., 1976; Latorre et al., 1985; Spotts et al., 2000; Rossi et al., 2009) and that infection from conidia overwintering on twig lesions only occur on severely affected pear trees.

Conidia from dormant mycelia in buds of pear trees have been considered the main source of primary inoculum for *V. nashicola* (Li, 1959; Yin and Yu, 1988). Ascospores of *V. nashicola* have also been considered an important source of primary inoculum in China (Lian et al., 2006) and Japan (Umemoto, 1990b).

*Venturia carpophila* overwinters as mycelia in lesions on fruit-bearing 1-year-old twigs (Scherm et al., 2008; Lalancette et al., 2012), and the conidia produced on these lesions act as the primary inoculum. Pseudothecia of *V. carpophila* were found in overwintering apricot leaves only in a severely affected orchard in Australia (Fisher, 1961). However, the epidemiological role of the teleomorph in the *V. carpophila* disease cycle remains unknown (Lalancette et al., 2012).

On pecan trees, *F. effusum* overwinters mainly on the surface of twigs and nuts as stromata, which sporulate profusely in early spring (Demaree, 1924). The most important inoculum sources of *F. oleagineum* are the infected leaves remaining in the olive canopy (Graniti, 1993; Viruega et al., 2013), because the fungus does not produce conidia on fallen, scabbed leaves (Viruega et al., 2013).

Little is known about the inoculum sources of *F. eriobotryae*. The fungus probably oversummers (its host, loquat, blooms in autumn, develops fruit in winter, and ripens in early spring) in lesions on branches and leaves and in mummified fruits (González-Domínguez et al., 2014a). However, the ability of the fungus to sporulate on these potential inoculum sources and their epidemiological role have never been studied.

#### Pseudothecia formation and ascospore maturation

For *V. inaequalis* and *V. nashicola*, light enhances pseudothecial production; pseudothecia formation is significantly reduced when the leaves overwinter in darkness (Table 3; Hirst and Stedman, 1962; Lian et al., 2006). Leaves overwintering in soil developed abnormal *V. inaequalis* pseudothecia, whereas those exposed weekly to 20 min of light produced normal ones (Holz, 1937). For both pathogens, moisture plays a key role. For *V. inaequalis*, rain is necessary for the growth of mycelium into the leaf lamina and for initiaion of the ascigerous stage (Machardy, 1996). Pseudothecia developed at low rates during dry periods and matured rapidly during rain periods (Wilson, 1928); however, continous wetness delayed ascospore maturation. Similarly, continuous wetness prevented pseudothecial development by *V. nashicola* (Lian et al., 2006). Pseudothecia formation and ascospore maturation occurred under a wide range of temperatures for both pathogens (O'Leary and Sutton, 1986; Lian et al., 2006).

#### Ascospore discharge and dispersal

Environmental requirements for ascospore discharge have been frequently studied for *V. inaequalis* (Table 3). For *V. inaequalis, V. pyrina*, and *V. nashicola*, ascospores are mainly discharged during or following rain events (Latorre et al., 1985; Umemoto, 1990a; Spotts and Cervantes, 1994; Machardy, 1996; Villalta et al., 2001; Eguchi and Yamagishi, 2007; Lian et al., 2007; Rossi et al., 2009). Villalta et al. (2001) captured 90% of *V. pyrina* ascospores during rain events, and Rossi et al. (2009) observed that at least 1.2 mm of rain was neccesary for ascopore ejection.

For *V. nashicola*, Lian et al. (2007) observed that 10 s of wetness was sufficient for ascospore discharge. For *V. inaequalis*, 0.0025 mm of rain may result in ascospore ejection, but >0.2 mm of rain is usually necessary to capture ascospores in traps (Machardy, 1996). Although some papers have reported the trapping of a few ascospore in periods without rain (Machardy, 1996), the trapping of many ascospores in the early morning following heavy dew at night has been reported only in Norway (Stensvand et al., 1998).

In general, a daily periodicity of ascospore discharge has been observed for *V. pyrina* (Latorre et al., 1985; Villalta et al., 2001), *V. nashicola* (Eguchi and Yamagishi, 2007), and *V. inaequalis* (Machardy, 1996). In all of these cases, most of the ascospores were trapped between 6:00 and 18:00 h.

Pseudothecia are able to eject ascospore to a height of 8 mm for *V. nashicola* (Umemoto, 1990a) and 5–13 mm for *V. inaequalis* (Aylor and Anagnostakis, 1991). After ejection, ascospores of both pathogens are dispersed by wind. Umemoto (1990a) was able to sample air-borne ascospores of *V. nashicola* to a distance of 10 m from the inoculum source. In the case of *V. inaequalis*, ascospores have been captures as far as 45 m from the inoculum source (Holb et al., 2004b).

#### Ascospore germination and infection

Ascospore germination has been studied for *V. inaequalis* and *V. nashicola* (Table 3), and had similar temperature requirements for the two species. The ascospores germinate between 5 and 30°C (germination of *V. inaequalis* has been observed at 0.5°C), with the optimum at 15–25°C (Louw, 1948; Boric, 1985; Lian et al., 2007). Ascospores begin to germinate after 2–3 h when the temperature is optimal; at 10°C, *V. inaequalis* germinated after 3 h of wetness and *V. nashicola* after 6 h of wetness (Boric, 1985; Lian et al., 2007).

Keitt and Jones (1926) were the first to conduct a controlled-environment experiment concerning the minimum number of hours of wetness required for infection by *V. inaequalis* ascospores. This information was then used by Mills (1944) to develop a chart representing the minimum hours of wetness for light, moderate, and severe infection. Although this publication is considered a milestone in plant pathology, many reports (reviewed by Machardy and Gadoury, 1989) have found that the chart requires modification. Infection by ascospores requires approximately 3 h less than the minimum proposed by Mills, i.e., it requires 5 h of wetness at 20°C and 8 h of wetness at 12° or 25°C. Therefore, Machardy and Gadoury (1989) proposed a new curve that describes the minimum hours of wetness necessary for infection at any temperature. Stensvand et al. (1997) subsequently modified the infection curve for temperatures between 2 and 8°C.

Villalta et al. (2000) reported that infection by *V. pyrina* ascospores was similar to that reported for *V. inaequalis* (Machardy and Gadoury, 1989) at temperatures below 10°C and at 25°C. In the optimal temperature range of 20–25°C, however, infection by *V. pyrina* ascospores required 9 h of wetness rather than the 5 h required by *V. inaequalis*.

#### Production of conidia

*Venturia inaequalis, V. nashicola and Fusicladium oleagineum* are able to sporulate at temperatures from 5 to 25°C (Table 3). *V. pyrina* can sporulate at temperatures between 5 and 28°C, which are the only temperatures tested for this species (Ben-Yephet, 1977), whereas *V. carpophila* can also sporulate at 30°C (Lalancette et al., 2012). The highest sporulation rate occurred from 15 to 20°C for all species, except for *F. eriobotryae*, which produced the most conidia at 5–10°C (Marras, 1963).

**Figure 4:**
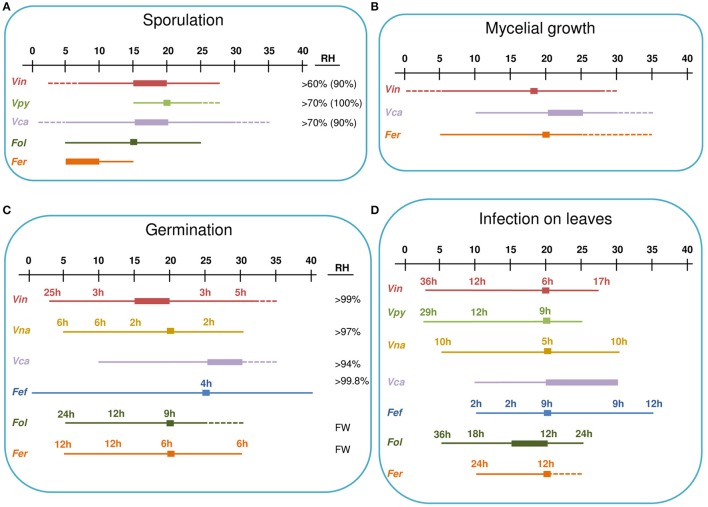
Environmental requirements of *Venturia* spp. for sporulation **(A)**, mycelial growth **(B)**, germinacion **(C)**, and leaves infection **(D)**. A temperature scale from 0 to 40°C is indicated at the top of each panel. Thin lines indicate the temperature at which the different processes occur for each species. Thick lines indicate optimal temperatures. Dotted lines indicate temperatures that are known not to support the process based on experimental evidence. Numbers indicate the hours of wetness necessary at each temperature. For sporulation **(A)**, the RH range in which the process can occur is indicated, with the optimal RH in brackets. For conidial germination **(C)**, the RH range in which the process can occur is indicated; FW indicates that free water is required for germination. Fef, *Fusicladium effusum*; Fer, *Fusicladium eriobotryae*; Fol, *Fusicladium oleagineum*; Vca, *Venturia carpophila*; Vin, *Venturia inaequalis*; Vna, *Venturia nashicola*; Vpy, *Venturia pyrina*.

*Venturia inaequalis* was able to sporulate between 60 and 100% RH, with the optimum at 90% RH (Studt and Weltzien, 1975). *V. carpophila* and *Fusicladium oleagineum* sporulated at <70% RH (Lawrence and Zehr, 1982; Obanor, 2006). For *F. oleaginum*, sporulation at 70% RH was <50% of the maximum observed under continuous wetness. Under optimal conditions, *V. carpophila* sporulation was highest after 72 h of incubation, whereas *F. oleaginum* sporulation still increased after 14 days of incubation at 100% RH (Lawrence and Zehr, 1982; Obanor, 2006; Lalancette et al., 2012; Figure 4).

#### Dispersal of conidia

Spore sampling studies have been conducted for several species (Table 3). These studies consistently show that conidia of *Venturia* spp. are mainly collected during or soon after rain events.

*Venturia* conidiophores (which are short and blunt) do not favor the removal of conidia by wind. Conidia of *V. inaequalis* were dislodged from dry, sporulating lesions by strong air currents only; the addition of a drop of water, however, caused conidiophores to swell and conidia to dislodge almost immediately (Frey and Keitt, 1925). In several spore-sampling studies in orchards, conidia were trapped from the air, frequently in low numbers, only during rainy weather; in only a few cases were high numbers of *V. inaequalis* conidia trapped during dry weather (Keitt and Jones, 1926; Gupta and Lele, 1980), with a diurnal periodicity and a peak in the afternoon (Hirst and Stedman, 1961). Machardy (1996) hypothesized that the release of conidia on dry days was triggered by the moisture provided by dew. A steep dispersal gradient was observed for *V. inaequalis* conidia, with few conidia sampled at >10 m from the inoculum source (Wiesmann, 1932).

For *V. pyrina*, conidia were also sampled from the air in periods with rain (Kienholz and Childs, 1937). The steep dispersal gradient for *V. nashicola* conidia (maximum dispersal distance = 8 m) suggests that these conidia are splash dispersed (Umemoto, 1990a).

*Venturia carpophila* and *F. effusum* conidia were traditionally considered both wind and splash dispersed (Gottwald, 1982, 1983; Gottwald and Bertrand, 1982; Latham, 1982; Lawrence and Zehr, 1982) because they were sampled from the air during several dry periods without rain (Gottwald and Bertrand, 1982; Latham, 1982; Lawrence and Zehr, 1982). In controlled-environment experiments, *V. carpophila* and *F. effusum* conidia were mainly dispersed in periods with a rapid decrease of RH and light (Gottwald and Bertrand, 1982; Gottwald, 1983). Latham (1982) observed a marked diurnal periodicity for *F. effusum* conidial dispersal, with a peak at 12:00 h, concomitant with decreasing humidity.

The role of rain dispersal for *V. carpophila* was re-evaluated by Lan and Scherm (2003). In a 4-year study, air-borne conidia contributed little to fruit scab in comparison to water-borne conidia; exclusion of splashing conidia decreased disease severity by >90%, and runoff of water from the twig to the fruit via the peduncle also contributed to scab development. Bock et al. (2011) showed that *V. carpophila* lesions are not uniformly distributed on the peach fruit surface, i.e., most lesions develop near the peduncle.

Conidia of *F. oleagineum* were mainly dispersed by rain, with a low degree of wind dissemination in the absence of rain when RH was high (Lops et al., 1993). *Fusicladium oleagineum* conidia were dispersed near the inoculum source (<10 m) with a linear and positive relationship between rainfall and numbers of conidia dispersed (Viruega et al., 2013). De Marzo et al. (1993) observed that the psocopteran *Ectopsocus briggsi* helps spread *F. oleagineum* conidia by carrying them on its body surface or by allowing them to pass without damage through its alimentary canal.

Dispersal of *F. eriobotryae* conidia was also closely associated with rain. More than 90% of the conidia were collected during rainy periods, and 0.2 mm of rain was sufficient to trigger a dispersal event (González-Domínguez et al., 2014b). A strong aggregation of loquat scab lesions between and within loquat trees also confirmed that *F. eriobotryae* conidia were mainly splash dispersed (Madden, 1992; González-Domínguez et al., 2014b).

#### Germination of conidia

Environmental effects on conidial germination have been studied *in vitro* for several species (Table 3 and Figure 4). Conidia of all of these species are able to germinate at temperatures between 10 and 30°C, with the exception of *F. oleagineum*, whose conidia did not germinate at temperatures >25°C (Obanor et al., 2007). Germination at 5°C occurs in all of the species in which it has been tested, i.e., *V. inaequalis, V. nashicola, F. effusum, F. oleagineum*, and *F. eriobotryae* (Figure 4). Temperatures >30°C have been tested only for *V. inaequalis* and *F. effusum*, whose conidia were able to germinate at 32 and 40°C, respectively (Converse, 1956; Boric, 1985). Optimal germination temperatures are close to 20°C for all of the species, except for *V. carpophila* and *F. effusum*, for which the optimum was 25°C.

Conidia of *V. inaequalis, V. nashicola, V. carpophila*, and *F. effusum* germinate at 94–99% RH, but germination was higher in free water (Converse, 1956; Lawrence and Zehr, 1982; Machardy, 1996; Li et al., 2003). Conidia of *F. oleagineum* and *F. eriobotryae* germinated only in free water (Obanor et al., 2007; González-Domínguez et al., 2013). In free water and at optimal temperatures, *V. inaequalis, V. nashicola*, and *F. effusum* began to germinate after 3, 2, and 4 h, respectively, whereas *F. oleagineum* and *F. eriobotryae* required 9 and 6 h, respectively. At 10°C in free water, *V. inaequalis* and *V. nashicola* began to germinate after 3 and 6 h, respectively, whereas *F. oleagineum* and *F. eriobotryae* required at least 12 h (Figure 4; Machardy, 1996; Li et al., 2003; Obanor et al., 2007; González-Domínguez et al., 2013).

#### Infection by conidia

The effect of environment on conidial infection has been studied for most of the *Venturia* spp. considered in this review (Table 3 and Figure 4). For *V. carpophila*, laboratory experiments have not been performed, and only general requirements were mentioned by Scherm and Brannen (2004). Mills and Laplante (1954) stated that *V. inaequalis* conidia were able to cause infection in two-thirds of the time required by ascospores. Subsequent laboratory and field studies have been reviewed by Machardy and Gadoury (1989). For conidial infection, the latter authors used the results of Schwabe (1980) to develop curves for minimum requirements of temperature and wetness duration for infection. The curve developed with data from laboratory studies was similar in shape to the Mill's curve but the time required to infect was greater for the Machardy and Gadoury curves than for the Mills curves.

All *Venturia* spp. are able to infect leaves at temperatures from 10 to 25°C, except for *F. eriobotryae*, which was unable to infect loquat plants at 25°C (Figure 4). Infection at 5°C was documented for *V. inaequalis, V. pyrina, V. nashicola*, and *F. oleagineum. V. nashicola, V. carpophila*, and *F. effusum* caused infection at 30°C, and *F. effusum* caused infection at 35°C (Figure 4).

**Figure 5:**
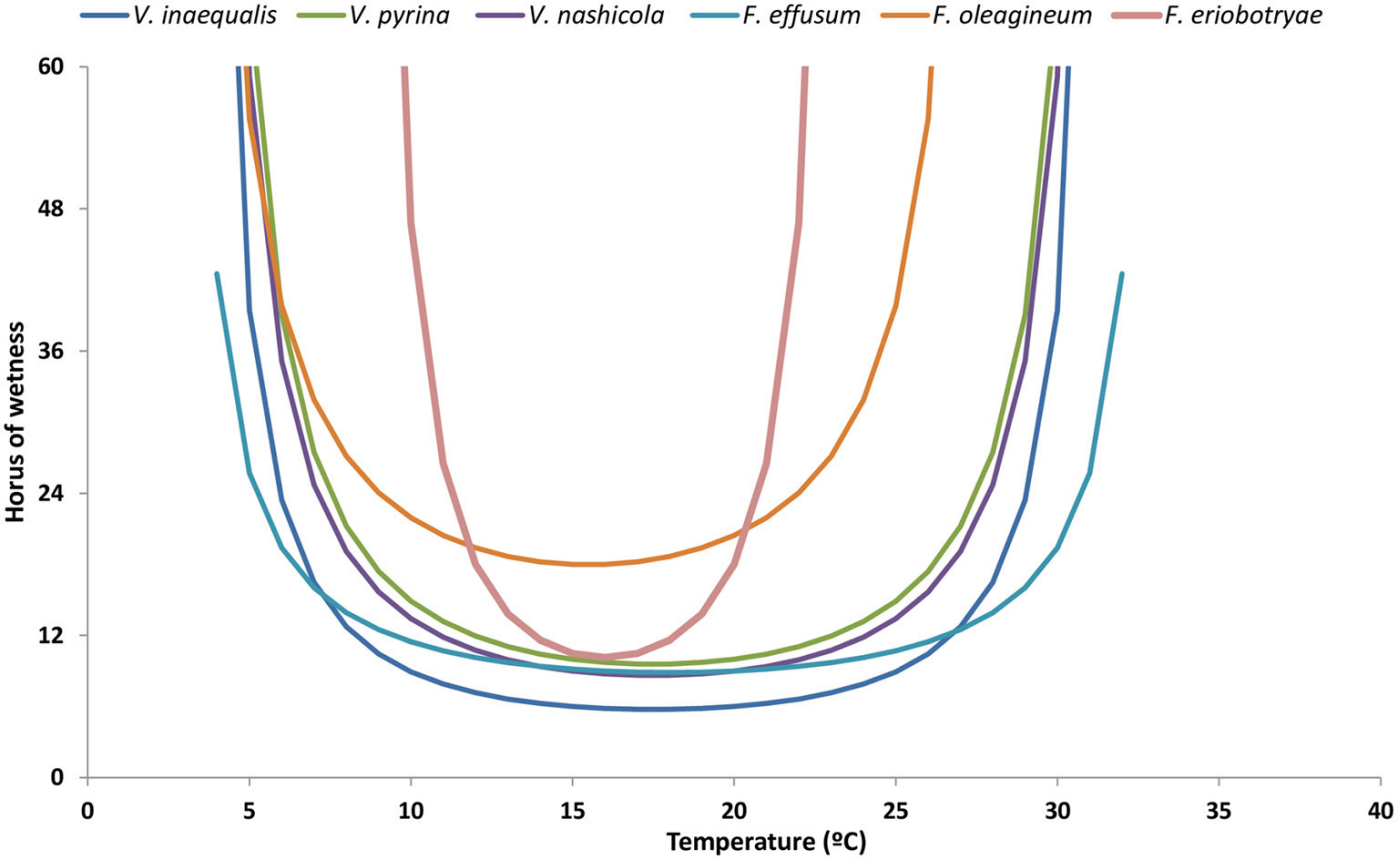
Minimum requirements of temperature and hours of wetness for conidial infection by *Venturia* spp. The requirements have been modeled by using the equation of Magarey et al. (2005). For each species, the maximum, minimum, and optimal temperatures for infection, and the minimum hours of wetness required are those indicated in Table 4.

For all of the species considered in this review, the optimal temperature for infection is 20°C, but there are differences in the minimum number of hours with high humidity or wetness required for infection (Figure 4). At 20°C and under continuous wetness, *V. nashicola* and *V. inaequalis* are able to infect within 5 and 6 h after inoculation, respectively; *V. pyrina* and *F. effusum* require 9 h, and *F. oleagineum* and *F. eriobotryae* only cause infection after 12 h of continuous wetness. At 10°C, the number of hours of continuous wetness required for infection ranged from 10 to 12 h for *V. inaequalis, V. pyrina*, and *V. nashicola*, 18 for *F. oleagineum*, and 24 for *F. eriobotryae. F. effusum* had similar wetness requirements (>2 h) at 10–35°C (Gottwald, 1985). The equation of Magarey et al. (2005) showed a similar behavior for *V. inaequalis, V. pyrina, V. nashicola*, and *F. effusum*, in that all four species were able to cause infection with only a few hours of wetness under a wide temperature range; *F. eriobotryae* and *F. oleagineum*, in contrast, had stricter requirements for both wetness duration and temperature (Figure 5).

#### Mycelial growth

*Venturia inaequalis, V. carpophila*, and *F. eriobotryae* grow at 10–25°C (Lawrence and Zehr, 1982; Machardy, 1996; González-Domínguez et al., 2013). *Venturia inaequalis* and *F. eriobotryae* also grow at 5°C, but this temperature has not been tested for *V. carpophila. V. carpophila* is able to grow at 30°C (Lawrence and Zehr, 1982). No additional information exists in the literature (Table 3 and Figure 4).

#### Latency period

Specific experiments on the effect of environmental conditions on the length of latency period (i.e., the time from infection until the occurrence of sporulating lesions) have been conducted only for *V. inaequalis* and *F. oleagineum* (Table 3). This period is shorter for *V. inaequalis* (ranging from 8 days at 18.6°C to 17 days at 9°C) than for *F. oleagineum* (60 days at 6°C, 16 days at 16°C and >120 days at 25°C; Mills, 1946; Roubal et al., 2013). For *V. inaequalis*, incubation at low RH (60–70%) for >9 days lengthened the latency period and lesions did not develop on plants incubated at low RH (Tomerlin and Jones, 1982). For *F. oleagineum*, leaf age affects the length of the latency period in laboratory experiments; latency ranged from 31 days in young leaves to 64 days in old leaves (Viruega et al., 2011).

## Multivariate analysis of the epidemiological components

As part of the current review, a multiple correspondence analysis (CA) was performed on epidemiological components of seven *Venturia* spp. CA is a multivariate statistical method that makes it possible to represent contingency tables in a pictorial form (Savary et al., 1995). CA is based on a raw data matrix, in which the rows are the objects and the columns are the variables. In this case, the objects are the seven *Venturia* spp.—*V. carpophila, V. inaequalis, V. pyrina, V. nashicola, F. effusum, F. eriobotryae*, and *F. oleagineum*—for which epidemiological information exists for seven qualitative variables and eight quantitative variables (Table 4).

**Table 4:** Epidemiological components of seven *Venturia* spp. used for three kinds of correspondence analysis (CA1, CA2, and CA3).

***Venturia* species**	**Climatic zones**^**a**^			**Sexual phase**	**Deciduous tree^b^**	**Ascospore infection**^**c**^				**Conidial infection**^**c**^				**Free water for conidial germination^d^**	**Wind dispersal of conidia**
	**C**	**ST**	**T**			**TM**	**Tm**	**To**	**Wm**	**TM**	**Tm**	**To**	**Wm**		
*F. effusum*	Y	Y	Y	N	N	0	0	0	0	37	7	20	9	N	Y
*F. eriobotryae*	N	Y	N	N	N	0	0	0	0	25	7	20	18	Y	N
*F. oleagineum*	N	Y	Y	N	N	0	0	0	0	28	3	15	18	Y	N
*V. carpophila*	N	Y	Y	Y	Y	0	0	0	0	30	5	18	6	N	Y
*V. inaequalis*	Y	Y	Y	Y	Y	30	4	20	6	32	3	20	6	N	Y
*V. nashicola*	N	N	N	Y	Y	30	5	20	6	32	3	20	12	N	N
*V. pyrina*	Y	Y	Y	Y	Y	30	1	20	9	32	3	22	10	N	Y
Average						30	3	20	7	31	4	19	11		
SD						0	2	0	2	4	2	2	5		
CA 1^e^	X	X	X	X	X	X	X	X	X	X	X	X	X	X	X
CA 2	–	–	–	X	X	X	X	X	X	X	X	X	X	X	X
CA 3	–	–	–	–	–	–	–	–	–	X	X	X	X	X	X

a *Climatic zones are: C, cold; ST, subtropical; T, tropical. These zones were proposed by Kottek et al. (2006) and are shown in Figure 2*.

b *Y and N indicate that the host is or is not deciduous*.

c *TM, maximum temperature for infection; Tm, minimm temperature for infection; To, optimal temperature for infection; Wm, minimum hours of wetness for infection*.

d *Y indicates that the species can germinate only in free water; N indicates that the species can germinate at <100% RH*.

e *For CA1, CA2, and CA3, X indicates the components used in each analysis*.

In our case, qualitative variables (e.g., the known presence of the sexual stage in nature) were classified using Yes or No; quantitative variables (e.g., optimal temperature for conidial infection) were ranked as high, medium, or low based on the average ± standard deviation (SD) of each data set. For example, the maximum temperature for conidial infection (TM) of the seven *Venturia* spp. ranged from 25 to 37°C, with an average of 31°C and a SD of 4. Thus, *Venturia* spp. in which the maximum temperature for infection was TM ≤ 27°C (i.e., 31–4) were classified as low, those with 27 > TM < 35°C were classified as medium, and those with TM ≥ 35°C were classified as high (Table 4).

The data matrix of Table 4 was used to perform CA with two dimensions (D1 and D2) using the multiple correspondence analysis procedure of SPSS (ver. 23; SPSS Inc.). Three analyses were performed: (i) with all components (CA1); (ii) with all components except those concerning the distribution in different climate types (CA2); and (iii) with only those components concerning the asexual stage (CA3). These analyses accounted for 80.0, 88.5, and 98.2% of data variance, respectively (Table 5).

**Table 5:** Statistics of three correspondence analyses (CA1, CA2, and CA3) performed for seven *Venturia* species with the data in Table 4.

**Analysis^a^**	**Dimension**	**Variance accounted for**		
		**Total^b^ (eigenvalue)**	**Inertia**	**% of Variance**
CA1	1	7.99	0.53	53.3
	2	3.99	0.27	26.7
	Total	11.99	0.80	80.0
CA2	1	7.63	0.64	63.6
	2	2.99	0.25	24.9
	Total	10.62	0.89	88.5
CA3	1	3.65	0.61	60.9
	2	2.24	0.37	37.3
	Total	5.89	0.98	98.2

a *CA1, CA2, and CA3 are the three analyses performed with different combinations of epidemiological components of seven Venturia spp. as indicated in Table 4*.

b *The magnitudes of the eigenvalues indicate the discriminating abilities of the dimensions*.

**Figure 6:**
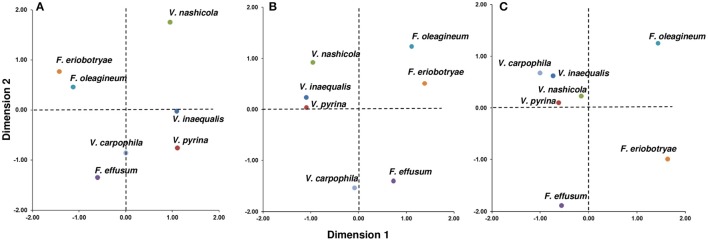
Distribution of seven *Venturia* spp. based on the environmental requirements of the pathogens and the biological characteristics of the pathogens and their hosts. **(A)** Joint plot of the correspondence analysis performed with 15 components related to climate and epidemiological variables of the sexual and asexual phase (CA1 in Table 4); **(B)** Joint plot of the correspondence analysis performed with 12 components related to epidemiological variables of the sexual and asexual phase (CA2 in Table 4); **(C)** Joint plot of the correspondence analysis performed with six components related to epidemiological variables of the asexual phase (CA3 in Table 4).

Overall, these analyses separated the *Venturia* spp. into two main groups when the seven species were plotted on the D1-D2 space (Figure 6). The first group contained *V. inaequalis, V. pyrina, V. nashicola*, and *V. carpophila*, and the second group contained *F. oleagineum* and *F. eriobotryae*, with F. *effusum* having an intermediate position depending on the CA analysis.

In CA1 (Figure 6A), grouping of the species was mainly based on the presence/absence of the sexual stage, infection of deciduous vs. non-deciduous trees, and the thermal and moisture requirements for ascosporic infection. These variables had the most influence (i.e., had high discrimination values, DVs) in D1, which accounted for 53.3% of the variance in the dataset (Table 5); the DV was >0.829 for these variables (Table 6). In CA1, *V. nashicola* was far from the other species in D2 (Figure 6A), mainly because of its different distribution among climate types (presence/absence of the species in tropical and subtropical climates had DVs = 0.514 and 0.637, respectively; Table 6); the presence/absence of the species in tropical and subtropical climates together with wind dispersal of conidia were the most influential variables in D2 (Table 6). This result may be biased by the distribution of *V. nashicola* being restricted to China, Japan, South Korea, and Taiwan, a restriction that may be caused more by quarantine measures by other countries than by differences in environmental requirements. This inference is supported by CA2, in which the pathogen distribution among climates was not considered. In CA2 (Figure 6B), the species were grouped mainly based on the presence/absence of the sexual stage and environmental requirements for ascosporic infection in D1 (Table 6), which accounted for 63.6% of the variance (Table 5). In CA3 (Figure 6C), the grouping was mainly determined by moisture conditions for conidial germination, infection, and dispersal in D1 (Table 6), which accounted for 60.9% of the variance (Table 5). The conidia of *V. inaequalis, V. pyrina, V. nashicola*, and *V. carphophila* are similar in that they require only a few hours of wetness (6–12 h) to infect and are capable of being dispersed by wind if dislodged by rain or dew. *Fusicladium oleagineum* and *F. eriobotryae* require longer periods of wetness to infect (>18 h), have lower maximum temperatures for infection (25–28°C), and have conidia that germinate only in the presence of free water. In CA3, *F. oleagineum* and *F. eriobotryae* had similar scores in D1, but they were far apart in D2 (Figure 6C) because of different temperature requirements for conidial infection (Tables 4, 6). Both *F. effusum* and the *Venturia* group can infect with <12 h of wetness and have wind-dispersed conidia that do not require free water to germinate; however, the temperature requirements for conidial infection differ between *F. effusum* and the *Venturia* group.

**Table 6:** Discrimination values (DVs) of the epidemiological components used in different correspondence analyses (CA1, CA2, and CA3) and dimensions (D1 and D2).

**Epidemiological component**		**CA1**^**a**^		**CA2**		**CA3**	
		**D1**	**D2**	**D1**	**D2**	**D1**	**D2**
Climatic zones^b^	Cold	0.214	0.377	–^f^	–	–	–
	Subtropical	0.151	0.514	–	–	–	–
	Tropical	0.022	0.637	–	–	–	–
Sexual phase		0.830	0.001	0.868	0.010	–	–
Deciduous tree^c^		0.830	0.001	0.868	0.010	–	–
Ascospore infection^d^	TM	0.829	0.079	0.824	0.119	–	–
	Tm	0.830	0.330	0.824	0.147	–	–
	To	0.829	0.079	0.824	0.119	–	–
	Wm	0.830	0.330	0.824	0.147	–	–
Conidial infection^d^	TM	0.459	0.353	0.478	0.340	0.460	0.884
	Tm	0.440	0.202	0.468	0.603	0.227	0.829
	To	0.212	0.035	0.206	0.253	0.343	0.260
	Wm	0.653	0.169	0.627	0.346	0.974	0.239
Free water^e^		0.652	0.151	0.623	0.303	0.942	0.007
Wind dispersion		0.214	0.742	0.197	0.591	0.709	0.020

a *CA1, CA2, and CA3 are the three analyses performed with different combinations of epidemiological components of seven Venturia spp. as indicated in Table 4*.

b *Climatic zones were proposed by Kottek et al. (2006) and are shown in Figure 2*.

c *Whether or not the host is deciduous*.

d *TM, Maximum temperature for infection; Tm, minimum temperature for infection; To, optimal temperature for infection; Wm, minimum hours of wetness for infection*.

e *Whether conidia can germinate only in free water*.

f *Indicates that this epidemiological component was not included in the analyses, as indicated in Table 4*.

The presence of two main groups of species probably reflects pathogen adaptation to host ecophysiology. This hypothesis is supported by the monophyly of the genus *Venturia* (Ishii and Yanase, 2000; Beck et al., 2005; Gladieux et al., 2010a; Bowen et al., 2011). *F. eriobotryae* and *F. oleagineum* are both pathogens of Mediterranean plants (loquat and olive, respectively). They are adapted to a warm and dry climate in which the low annual rainfall is distributed mainly in autumn and spring (Csa climate class; Graniti, 1990; Kottek et al., 2006). The absence of the teleomorph in nature may be related to the mild winter temperatures, which can be survived without a quiescent stage. In these fungi, dispersal of conidia occurs only during rain events, perhaps because conidia dispersed in water have a higher probability of germinating and causing infection. The requirement of free water for conidial germination and long periods of wetness for conidial infection may also be adaptations to a dry climate. These requirements would prevent the initiation of an infection cycle in the driest periods of the year. *F. eriobotryae* and *F. oleagineum* also have low mycelium growth rates and long latent periods. The evergreen habitus of their hosts means that these species do not require short infection cycles, because the trees are susceptible throughout the year and because the inoculum may survive on the tree during the season in which environmental conditions are not suitable for sporulation and infection.

*Venturia* spp. that attack deciduous trees, in contrast, require a sexual stage to survive the winter, when there is no host tissue to be infected and temperatures are low. Their conidia can germinate in the absence of free water, and infection requires fewer hours of wetness, especially in the case of *V. inaequalis* and *V. nashicola*. Together, these factors result in relatively short infection cycles, with a higher probability of occurrence. In this case, the deciduous habitus of the host makes the occurrence of infection obligatory, because the main inoculum source for the next season will be the fallen, scabbed leaves, even though overwintering in twig lesions and/or buds is possible.

## Conclusions and implications for scab management

This review has considered several important aspects of the phylogeny, host range, and life cycle of *Venturia* spp. affecting fruit trees. These species are responsible for some of the most important diseases of their hosts.

*Venturia* spp. affecting fruit trees are highly host-specific, as indicated by the general failure to obtain infection by cross-inoculation (Menon, 1956; Raabe and Gardner, 1972; Ishii and Yanase, 2000; Stehmann et al., 2001; Le Cam et al., 2002; Chevalier et al., 2004; Sánchez-Torres et al., 2007a, 2009; Abe et al., 2008). This conclusion partially contradicts some previous reviews of the genus *Venturia* (Sivanesan, 1977) and *Fusicladium* (Schubert et al., 2003), and has implications for the management of scab diseases in areas where different host species are grown, as is the case, for instance, in the Emilia-Romagna region of North Italy where apple, pear, cherries, and peaches are grown in close proximity. The inoculum produced in one crop cannot infect another crop, and management of a species of *Venturia* in one orchard has no effect on neighboring crops of other host species.

Although *Venturia* is one of the most famous and studied genera of plant pathogens, important gaps in understanding the life cycle still remain for some species. This is particularly unexpected for *V. pyrina* because of the worldwide importance of the crop and because the gaps involve key aspects of the life cycle, including pseudothecia formation, ascospore and conidia germination, and mycelial growth. The only work regarding dispersal of *V. pyrina* conidia was published in 1937 (Kienholz and Childs, 1937). In most cases, researchers have assumed that *V. pyrina* requirements are similar to those of *V. inaequalis*. This assumption, however, is not valid for ascosporic infection because ascospores of *V. pyrina* require more wet hours to infect (Figure 4). In the case of *V. carpophila*, specific experiments on the requirements for infection have never been performed, and this limits our ability to correctly manage the disease.

*Venturia* spp. can infect several parts of the host trees, but the main damage usually results from fruit infection, except for *F. oleagineum* affecting olive leaves, which can result in important economic losses. For this reason, fruit growers are generally risk-adverse to scab diseases and schedule a high number of fungicide applications to achieve a very high level of disease control. Machardy (1996) reported that USA apple growers schedule fungicide applications to achieve <1% of scabbed fruit at harvest. Similar thresholds are usually assumed for loquat, a high value fruit crop in Europe (E. Soler, personal communication). Current trends in disease management aim to avoid this high number of treatments, which involve risks to human health and the environment, and encourage the use of decision support systems (DSSs) (Rossi et al., 2012).

The Mills tables represent one of the first and better known DSSs and have been widely accepted by growers and advisors (Machardy, 1996). However, the use of these tables should be avoided for *Venturia* spp. other than *V. inaequalis*. For *V. pyrina, F. oleagineum, F. effusum*, and *F. eriobotryae* the Mills tables over-predict the number of scab infections because temperature and wetness requirements of these fungi are different from those of *V. inaequalis* (Figure 6; Gottwald, 1985; Villalta et al., 2000; Viruega et al., 2011; González-Domínguez et al., 2013). Moreover, the Mills tables can over-predict the number of infections even for apple scab (Machardy and Gadoury, 1989).

For some of the diseases considered in this review, epidemiological models have been developed to predict disease development (Table 7). Most of these models are simple and consider only one component of the pathogen life cycle, mainly ascospore maturation or infection. A main constraint of these models is that they have never been validated against independent data, i.e., model output (the prediction) has not been compared with a data set of real-world observations different from that used for model development (Rossi et al., 2010). Before these models are used in practical disease control, a robust validation with real data should be performed; the validation data should be obtained from different areas with different epidemiological conditions and for several years (Rossi et al., 2010).

**Table 7:** Characteristics of the epidemiological models developed for the *Venturia* spp. considered in this review.

***Venturia* species**	**Reference**	**Modeling approach**	**Sexual phase considered**	**Epidemiological components included^a^**	**Validation^c^**
*V. inaequalis*	Mills and Laplante, 1954	Empirical	1° and 2°	INF	Yes
	Xu et al., 1995	Mechanistic	1° and 2°	1°: DISP/INF//2°: DISP/INF/MORT	Yes
	Rossi et al., 2007	Mechanistic	1°	PSEUD MAT/ASC MAT/DISP/INF/INC/MORT	Yes
	Machardy and Gadoury, 1989	Empirical	1° and 2°	INF	Yes
	Beresford et al., 2004	Empirical	1°	DISP/INF/LAT	No
	Stensvand et al., 2005	Empirical	1°	ASC MAT	Yes
	Gadoury and Machardy, 1982	Empirical	1°	ASC MAT	No
*V. nashicola*	Li et al., 2007	Mechanistic	2°	DISP/INF/MORT	Yes
*V. pyrina*	Sobreiro and Mexia, 2000	Empirical	–^b^	INF	Yes
	Spotts et al., 2000	Empirical	1°	ASC MAT	No
*V. carpophila*	Lalancette et al., 2012	Empirical	2°	SPOR	Yes
*F. oleagineum*	Roubal et al., 2013	Empirical	2°	INF/LAT	No
	Viruega et al., 2011	Empirical	2°	INF	No
*F. eriobotryae*	González-Domínguez et al., 2014a	Mechanistic	2°	DISP/GERM/INF/MORT	Yes
*F. effusum*	Payne and Smith, 2012	Empirical	2°	INF	No

a *ASC MAT, Ascospore maturation; DISP, dispersion; GERM, germination; INC, incubation; INF, infection; LAT, latency; MORT, mortality; PSEUD MAT, pseudothecial maturation; SPOR, sporulation*.

b *Not specified*.

c *Indicates whether the model output has been compared with a data set of real-world observations different from that used for model development*.

In the case of *V. inaequalis*, mechanistic weather driven models have been developed for primary infections (Rossi et al., 2007) and for the whole life cycle (Xu et al., 1995). A mechanistic approach has also been used to develop epidemiological models for *V. nashicola* and *F. eriobotryae*, and these models consider most of the components of the life cycle (Li et al., 2007; González-Domínguez et al., 2014a). Several advantages have been previously reported for mechanistic vs. empirical models (Caffi et al., 2007; De Wolf and Isard, 2007; Rossi et al., 2010). Mechanistic models attempt to capture the full complexity of the pathogen life cycle and are generally considered to have greater explanatory ability than purely empirical models (De Wolf and Isard, 2007). A main advantage of the mechanistic models is that they can easily incorporate information from previous experiments regarding pathogen biology and epidemiology. Thus, this review should help researchers develop mechanistic models for those scab diseases that currently lack such models. Until such mechanistic models are available, however, the Magarey curves, developed in this review and which identify the requirements for infection, could be used as a starting point to predict infection risk, especially for *V. pyrina* and *F. effusum*.

## Author contributions

All the authors contributed to the writing of the manuscript. EG and VR performed the data analyses.

### Conflict of interest statement

The authors declare that the research was conducted in the absence of any commercial or financial relationships that could be construed as a potential conflict of interest.
